# Diosgenin Ameliorates Non-alcoholic Fatty Liver Disease by Modulating the Gut Microbiota and Related Lipid/Amino Acid Metabolism in High Fat Diet-Fed Rats

**DOI:** 10.3389/fphar.2022.854790

**Published:** 2022-04-25

**Authors:** Yuan Zhou, Ruoqi Li, Yingyi Zheng, Meiying Song, Shanshan Zhang, Yunxia Sun, Mengying Wei, Xiang Fan

**Affiliations:** ^1^ School of Basic Medical Sciences, Zhejiang Chinese Medical University, Hangzhou, China; ^2^ Department of Pharmacology and Department of Gastroenterology of the Second Affiliated Hospital, Zhejiang University School of Medicine, Hangzhou, China; ^3^ Key Laboratory of Neuropharmacology and Translational Medicine of Zhejiang Province, Zhejiang Chinese Medical University, Hangzhou, China

**Keywords:** diosgenin, non-alcoholic fatty liver disease, fecal metabolomics, gut microbiota, lipid metabolism, amino acid metabolism

## Abstract

Non-alcoholic fatty liver disease (NAFLD) is a metabolic disease closely associated with dietary habits. Diosgenin is abundant in yam, a common food and traditional Chinese medicine. The molecular mechanism of diosgenin on NAFLD has been preliminarily explored. However, the effect of diosgenin on metabolism and gut microbiota in NAFLD has not been reported. This study confirmed that diosgenin could suppress excessive weight gain, reduce serum levels of total cholesterol and triglycerides, and decrease liver fat accumulation in high-fat diet-induced NAFLD rats. Moreover, fecal metabolomics analysis suggested diosgenin improved abnormal lipid and amino acid metabolism. Bile acids, including lithocholic acid and ursodeoxycholic acid 3-sulfate that function as excretion, absorption, and transport of fats, were remarkably regulated by diosgenin. Aromatic amino acid and lysine metabolism was regulated by diosgenin as well. 16S rRNA gene sequencing analysis demonstrated that diosgenin restored gut microbiota disorder, especially *Globicatella, Phascolarctobacterium, Pseudochrobactrum, and uncultured_bacterium_f_Prevotellaceae* at the genus level. Additionally, these regulated bacterial genera showed significant correlations with lipid and amino acid metabolism-related biomarkers. This study further confirmed the significant effect of diosgenin on NAFLD, and provided a new perspective for the mechanism.

## 1 Introduction

Hepatic steatosis without significant alcohol consumption, monogenic hereditary disorders, long-time steatogenic medication use, or other secondary causes of hepatic fat accumulation is defined as Non-alcoholic fatty liver disease (NAFLD) (Chalasani et al., 2018). With NAFLD progress, simple steatosis has the potential to develop to non-alcoholic steatohepatitis even liver cirrhosis (Jingda Li et al., 2021). More remarkably, NAFLD has been proved to be a pathogenic factor of hepatocellular carcinoma (Kulik and El-Serag, 2019). Up to now, lifestyle interventions such as a reasonable diet and proper exercise are still the most basic and effective way for NAFLD treatments. For some severely overweight NAFLD patients, bariatric surgery could help them directly reduce fat in the liver and also lose weight. Though many drugs, such as farnesoid X receptor (FXR) agonists, thyroid hormone receptor *β* agonists, etc., are undergoing clinical evaluation, none has been approved (Petroni et al., 2021).

With the in-depth study of NAFLD, abnormal lipid/amino acid metabolism and related gut microbiota disorder have attracted enormous interest from researchers (Aron-Wisnewsky et al., 2020). Almost all lipids and lipid-like molecules, including fatty acids, oxidized fatty acids, triglycerides (TG), phospholipids, sphingolipids, and bile acids, are detected abnormal levels in NAFLD patients and animals (Masoodi et al., 2021). Liver biopsy samples from NAFLD patients provided direct evidence that both polyunsaturated fatty acids and saturated fatty acids were remarkably increased in the liver from NAFLD patients (Puri et al., 2007). Similarly, oxidized fatty acids represented by hydroxyeicosatetraenoic acids (HETE) and hydroxyoctadecadienoic acids (HODE) were observed significant content changes as well (Masoodi et al., 2021). Bile acids play roles in the excretion, absorption, and transport of fats and sterols in the intestine and liver. And bile acids can affect NAFLD *via* FXR signaling pathway (Jiao et al., 2018).In addition to lipids, amino acid metabolites are another class of biomarkers of NAFLD. Researchers have found that serum levels of branched-chain amino acids (BCAAs) and aromatic amino acids (AAAs) increased in people with liver fat accumulation. Also, many NAFLD model animals exhibited remarkable amino acid metabolism disorders (Ahmad et al., 2020). Gut microbiota change plays a critical role in lipid/amino acid metabolism(Aron-Wisnewsky et al., 2020; Zhu et al., 2021). Gut microbiota disturbance induced dysfunction of the gut-liver axis is indicated to promote the occurrence and development of NAFLD (Mu et al., 2021). BCAAs, AAAs and short-chain fatty acids (SCFAs) are regulated by gut microbiota in NAFLD (Masoodi et al., 2021). More dramatically, gut microbiota is indispensable for transformation processes, including deconjugation, dehydroxylation and oxidation of some bile acids in the gut (Funabashi et al., 2020). Researchers have found non-negligible bile acid and gut microbiota disorders in NAFLD patients (Chen et al., 2019).

Given the non-negligible roles of lipid/amino acid metabolism and related gut microbiota in NAFLD, much effort was made to target them for NAFLD amelioration. Functional foods have become one of the most concerning therapies because of their excellent effects and high security (Chen et al., 2021). Diosgenin is the aglycone of dioscin, both of which are abundant in yam (*Dioscorea oppositifolia* L.), a kind of food often appears on the dinner table of East Asians and also a traditional herb medicine in China. Dioscin could be hydrolyzed to diosgenin in mammalian body, and diosgenin could not be further metabolized (Li et al., 2019). Traditional Chinese medicine provides direction for research on functional foods and natural products based on abundant clinical practices (Zhou et al., 2021a; Zhou et al., 2021b). Yam, usually applied to treat digestive system diseases, reminds researchers of the potential function of diosgenin in metabolic disorders.

Diosgenin has been reported to exhibit considerable lipid-lowering effects in several lipid metabolism disorders such as obesity, hyperlipidemia, hypercholesterolemia, and atherosclerosis (Li et al., 2019; Wu and Jiang, 2019; Khateeb et al., 2021; Sun et al., 2021). Diosgenin could prevent NAFLD by AMP-activated protein kinase activation and FXR suppression (Cheng et al., 2018). In addition, diosgenin was able to improve the expression of lipolysis proteins, including p-AMPK, phospho-acetyl coA carboxylase, and carnitine acyl transferase1A, as well as inhibit expression of lipid synthesis-related proteins, including sterol regulatory element-binding protein 1c and fatty acid synthase (Fang et al., 2019; Khateeb et al., 2021). In the current study, we further confirmed the function of diosgenin in ameliorating high-fat diet-fed NAFLD rats and explored the changes in endogenous metabolites and intestinal microbiota through fecal metabolomics and 16S rRNA gene sequencing analyses. Our work could provide a more comprehensive and detailed understanding of the mechanism of diosgenin on NAFLD.

## 2 Materials and Methods

### 2.1 Materials and Methods

Diosgenin (purity above 98%, HPLC) was purchased from Beijing gersion Bio-Technology Co., Ltd. (Beijing, China). Simvastatin was purchased from Hangzhou MSD Pharmaceutical Co., Ltd. (Hangzhou, China). Tween 80 was purchased from Sigma-Aldrich (St Louis, United States). LC-MS grade methanol and acetonitrile were purchased from CNW Technologies GmbH (Duesseldorf, Germany). LC-MS grade ammonium acetate was purchased from Sigma-Aldrich (St Louis, United States). LC-MS grade ammonium hydroxide was purchased from Fisher Chemical (Waltham, United States) and ddH_2_O was purchased from Watsons (Hongkong, China). Total cholesterol (TC) and TG Kits were purchased from Jiancheng Institute of Biotechnology (Nanjing, China).

### 2.2 Animals and Treatments

The whole procedure of animal experiments was performed under standard laboratory conditions and approved by the Animal Ethics Committee of Zhejiang Chinese Medical University (ethical approval number: IACUC-20201214-10).

#### 2.2.1 Establishment of NAFLD Model

Forty-five specific-pathogen-free Sprague-Dawley rats (male, 6 weeks, 160 g–180 g) purchased from Vital River Laboratory Animal Technology Co., Ltd (Beijing, China) were adaptively fed for 1 week at first. Then ten rats were given ordinary feed, and thirty-five rats were fed with a high-fat diet (15% fat, 1% cholesterol, and 0.2% sodium cholate) for 4 weeks. Then two rats fed a regular diet and three rats fed a high-fat diet were randomly selected and sacrificed for liver observation, other rats were executed for blood collection through the orbital vein for serum TC and TG measurement.

#### 2.2.2 Grouping and Treatment

After the establishment of NAFLD model, eight normal diet-fed rats formed a normal control group (NG), and thirty-two high fat diet-fed rats were randomly divided into four groups: NAFLD model group (MG), low dose group (LG), high dose group (HG), and simvastatin group (SG), respectively. Diosgenin and simvastatin were dissolved in saline with 2% tween 80 for intragastric administration. LG (0.15 g/kg/d diosgenin), HG (0.3 g/kg/d diosgenin), and SG (4 mg/kg/d simvastatin) were treated for 8 weeks, while NG and MG were given vehicle in the same way. The dose of diosgenin was determined based on our previous work (Li et al., 2019). NG was given a normal diet during the entire administration period, while other groups were still given a high-fat diet. Food intake of all groups was recorded as well. All rats were weighed once a week and sacrificed after 8 weeks of treatment.

### 2.3 Serum Biochemical and Histopathological Analyses

Blood samples were collected from the abdominal aorta and anticoagulated by heparin sodium after fasting 12 h and the last administration. Plasma was obtained from each sample by centrifuging at 3000 rpm for 10 min at 4°C. The plasma levels of TC and TG were measured according to the Kits instruction.

Part of liver tissues were fixed in 10% formalin, dehydrated, and embedded in paraffin for hematoxylin and eosin (H&E) staining. The tissues were cut into 5 µM sections by microtome (RM2245, Leica, United States) and subsequently stained with H&E. The other part of liver tissues was applied for Oil Red O staining. The frozen liver tissues were cut into 6 μM thick sections using a microtome-cryostat (NX70, Thermo Fisher Scientific, United States), air-dried on glass slides, and then fixed with 10% formaldehyde solution for 10 min. Subsequently, the sections were rinsed with distilled water and soaked with 60% isopropanol. After that, sections were performed for Oil Red O staining and hematoxylin counterstaining. Both H&E and Oil Red O stained sections were captured with a microscope (Axio Observer 3, Zeiss, Germany).

### 2.4 Fecal Metabolomics

#### 2.4.1 Sample Collection and Preparation

Feces samples from NG, MG, and HG were harvested and froze quickly by liquid nitrogen at 1 h after the last administration and stored at −80°C for later use. 25 mg feces were mixed with extract solution (methanol: acetonitrile: water = 2: 2: 1, with isotopically-labelled internal standard mixture). Then the mixture was homogenized at 35 Hz for 4 min and sonicated for 5 min in the ice-water bath. The homogenization and sonication were repeated for 3 times. After 1 h incubation at −40°C and 15 min centrifugation at 12,000 rpm at 4°C, supernatant were harvested for LC-MS analysis. Quality control (QC) sample was prepared by mixing an equal aliquot of the supernatants from all samples.

#### 2.4.2 Sample Detection by UPLC-Q-TOF-MS

UPLC system (Vanquish, Thermo Fisher Scientific), UPLC BEH Amide column (2.1 mm × 100 mm, 1.7 μM, normal phase column), and Q Exactive HFX mass spectrometer (Orbitrap MS, Thermo) were adopted cooperatively for UPLC-Q-TOF-MS analysis. The mobile phase consisted of 25 mmol/L ammonium acetate and 25 ammonia hydroxide in water (pH = 9.75) (A) and acetonitrile (B). Elution was as follows: start with 5% solvent A and 95% solvent B for 30 s, decrease to 65% B at 7 min, decrease to 40% B at 8 min, solvent maintained for 1 min, returned to 95% B for 0.1 min and held for approximately 2.9 min. The auto-sampler temperature was 4°C, and the injection volume was 3 μl. The mass spectrometer applied ESI source, whose conditions were set as following: sheath gas flow rate as 30 Arb, Aux gas flow rate as 25 Arb, capillary temperature 350°C, full MS resolution as 60,000, MS/MS resolution as 7500, collision energy as 10/30/60 in NCE mode, spray Voltage as 3.6 kV (positive) or −3.2 kV (negative), respectively.

#### 2.4.3 Processing and Analysis of UPLC-Q-TOF-MS

UPLC-Q-TOF-MS raw data was preliminarily managed by following four steps: filtering deviation value, filtering missing value, filling missing value, and normalizing data (Dunn et al., 2011). Then SIMCA (V16.0.2, Sartorius Stedim Data Analytics AB, Umea, Sweden) was applied for principal component analysis (PCA) and orthogonal projections to latent structures discriminant analysis (OPLS-DA). Afterward, the statistical analysis combined with unit variables and multivariate variables was used for differential metabolites screening. *p*-value less than 0.05 (student’s t-test) and variable importance in projection greater than 1 (OPLS-DA model) were two indexes for screening. HMDB (http://www.hmdb.ca/) and KEGG (http://www.kegg.com/) provided necessary information about metabolites and their metabolic and/or synthetic processes (Zhou et al., 2018).

### 2.5 16S rRNA Gene Sequencing Analysis

Total genomic DNA from fecal samples was extracted by Tiangen Fecal Genomic DNA Extraction Kit (Beijing, China). After determining the quantity of extracted genomic DNA, the bacterial V3-V4 hypervariable regions of 16S rRNA were amplified by PCR. The forward primer (338F) was 5’-ACT​CCT​ACG​GGA​GGC​AGC​A-3’, and the reverse primer (806R) was 5’-GGACTACHVGGGTWTCTAAT-3’. Illumina Novaseq was applied for sequencing, then Base Calling was performed for Sequenced Reads, and the results were stored in FASTQ format files. The data were preprocessed for further analysis as follows: filtering merged Raw Tags to get high-quality Clean Tags by software Trimmomatic v0.33; identifying and removing chimeric sequences to get Effective Tags by software UCHIME v4.2. The data analysis method was described previously (Zhou Z. et al., 2021; Lin Li et al., 2021).

### 2.6 Statistical Analysis

All experimental data were presented as mean ± standard deviation (SD). Statistical analysis was performed by SPSS Statistics 22.0, and groups differences were evaluated by one-way analysis of variance (ANOVA) and LSD was applied for post-hoc test. *p* < 0.05 were considered significant.

## 3 Results

### 3.1 Diosgenin Decreased the Weight Gain and Mitigated Serum Levels of TC and TG in High-Fat Diet-Fed Rats

**FIGURE 1:**
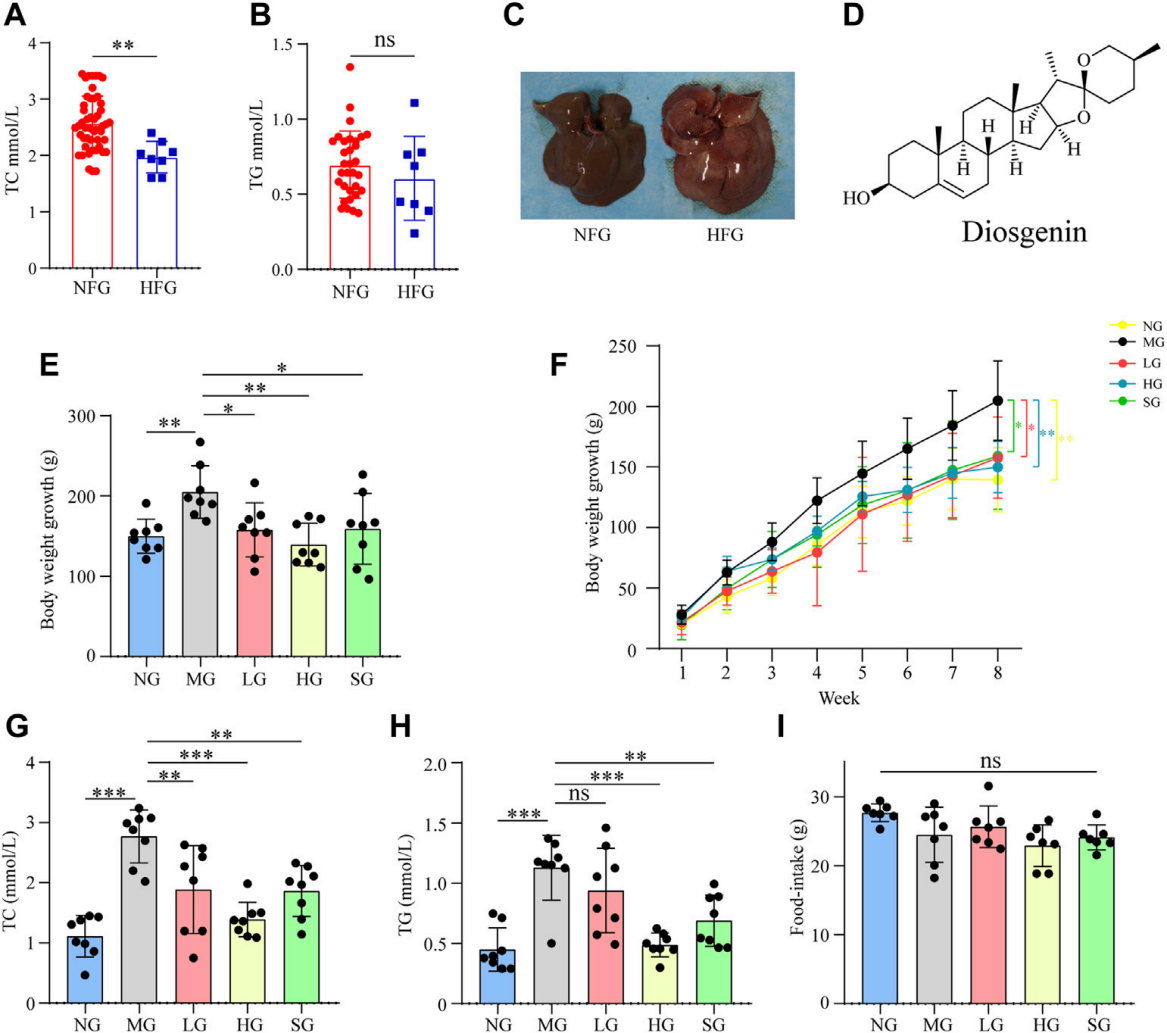
Impacts of high fat diet and diosgenin on body weight, serum biochemical parameters, and food-intake in SD rats, ***p < 0.001, **p < 0.01, *p < 0.05, ns: no significant difference **(A)** serum TC level in NFG (Normal feed for 4 weeks group) and HFG (high-fat diet-fed for 4 weeks group); **(B)** serum TG level in NFG and HFG; **(C)** macroscopic pictures of livers in NFG and HFG; **(D)** Chemical structure of diosgenin; **(E)** body weight growth over 8 weeks; **(F)** body weight growth curve in 8 weeks; **(G)** final serum TC level; **(H)** final serum TG level; **(I)** average food-intake in 8 weeks.

After 4 weeks of high-fat diet feeding, the serum level of TC (Figure 1A) was remarkably increased. While serum level of TG (Figure 1B) between high-fat diet-fed rats and normal diet-fed rats showed no significant difference. High-fat diet induced accumulation of fat in the liver (Figure 1C) indicated the reliability of the NAFLD model. Both diosgenin (Figure 1D) and simvastatin decreased the weight gain of high-fat diet-fed rats (Figures 1E,F). During 8 weeks of treatment, MG gained more weight than NG (*p* < 0.01) (Figure 1E). Compared to MG, other treatment groups decreased body weight significantly (Figure 1E). High-fat diet increased serum level of TC, and high dose of diosgenin exerted optimal down-regulation effect of TC (*p* < 0.001 compared to MG) (Figure 1G). As for TG, HG, and LG showed different results (Figure 1H). The serum level of TG in HG was obviously less than MG (*p* < 0.001), while the serum level of TG in LG and MG showed no significant difference (*p* > 0.05). As an antihyperlipidemic drug, simvastatin mitigated high serum levels of TC and TG as well (Figures 1G,H). It is worth mentioning that the food intakes of all groups showed no significant difference (Figure 1I). Thus, diosgenin played roles in weight, TC and TG regulations by an internal mechanism rather than food intake change.

### 3.2 Diosgenin Reduced Fat Accumulation in the Liver of High-Fat Diet-Fed Rats

**FIGURE 2:**
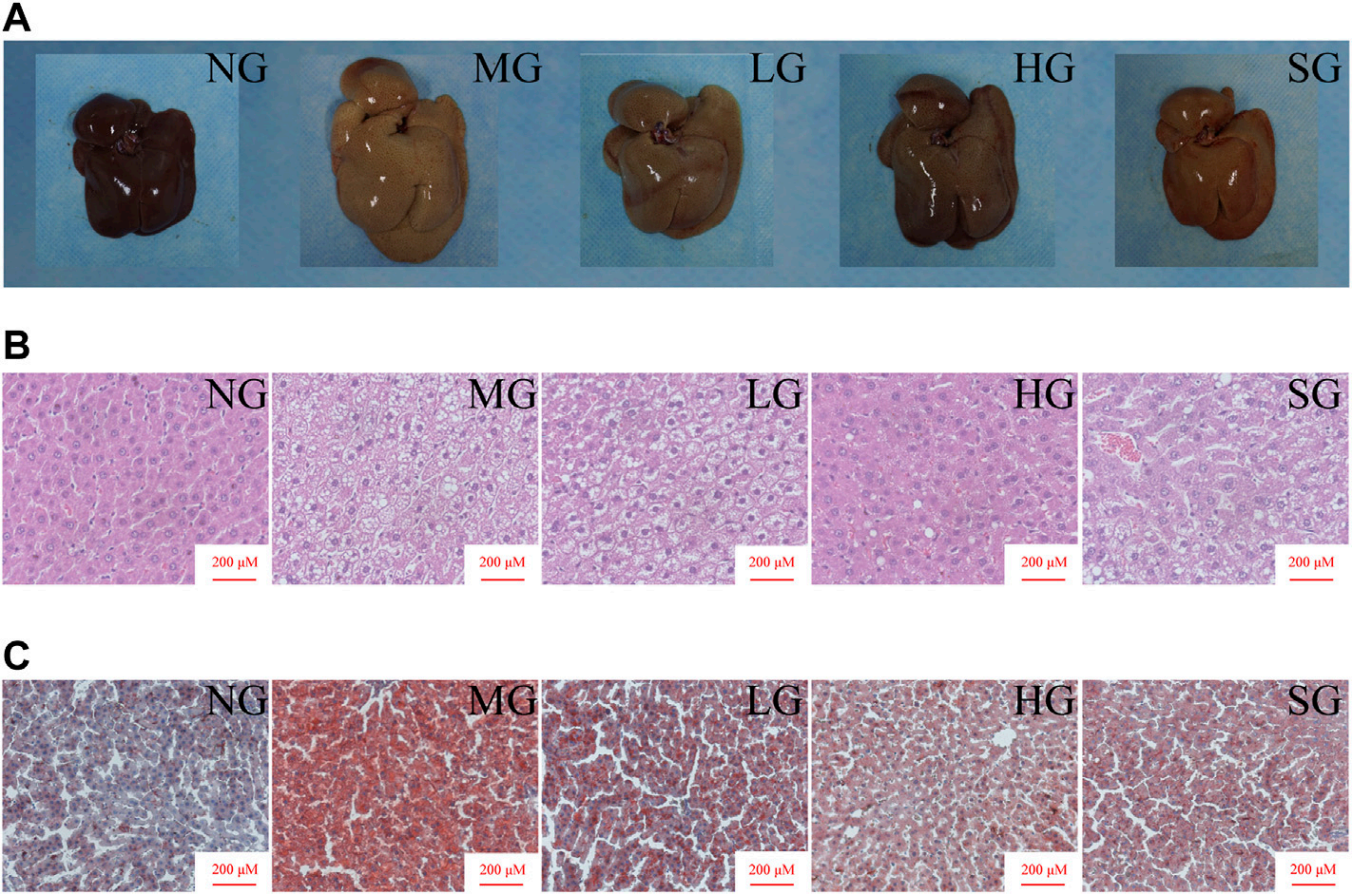
Impacts of diosgenin on livers from SD rats **(A)** macroscopic pictures of livers; **(B)** representative liver sections stained with H&E; **(C)** representative liver sections stained with oil red.

The liver size of MG was more significant than that of NG, and the liver color was yellow and greasy. Diosgenin alleviated these changes after 8 weeks of administration (Figure 2A). H&E staining showed apparent lipid accumulation in the hepatocytes filled with small vacuoles and necrosis in MG (Figure 2B). These hepatic steatosis and fat accumulation were mitigated in all treatment groups. Analogously, diffused and granular lipid depositions were observed in the liver from the MG by oil red O staining (Figure 2C). LG, HG, and SG groups markedly reduced lipid deposition in hepatocytes compared to MG. To summarize, diosgenin observably reduced fat accumulation in livers of NAFLD rats.

### 3.3 Fecal Metabolic Profile Change and Potential Metabolite Biomarkers Identification

**FIGURE 3:**
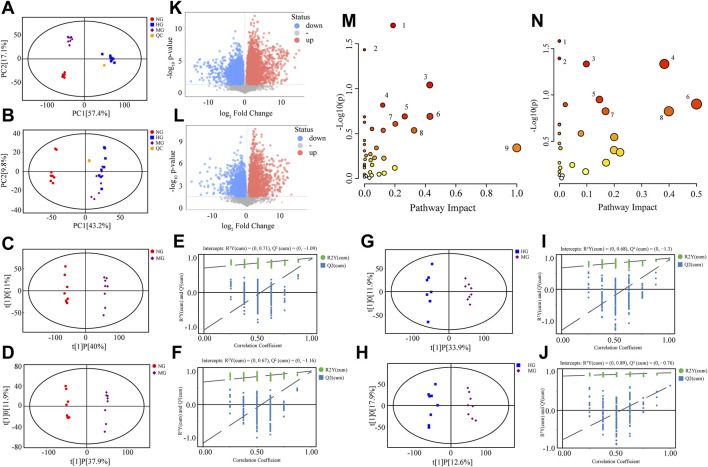
Metabolomics analysis of NG, MG, and HG. PCA score plots in positive mode **(A)** and negative mode **(B)**; OPLS-DA score plots for NG vs. MG in positive mode **(C)** and negative mode **(D)**; permutation test of OPLS-DA model for NG vs. MG in positive mode **(E)** and negative mode **(F)**; OPLS-DA score plots for HG vs. MG in positive mode **(G)** and negative mode **(H)**; permutation test of OPLS-DA model for HG vs. MG in positive mode **(I)** and negative mode **(J)**; volcano plots for NG vs. MG in positive mode **(K)** and negative mode **(L)**; **(M)** overview of metabolic pathways changed by high fat diet: 1. Lysine degradation, 2. Linoleic acid metabolism, 3. Taurine and hypotaurine metabolism, 4. Sphingolipid metabolism, 5. Glycerophospholipid metabolism, 6. Arachidonic acid metabolism, 7. Steroid biosynthesis, 8. Tyrosine metabolism, 9. Ubiquinone and other terpenoid-quinone biosynthesis; **(N)** overview of metabolic pathways regulated by diosgenin: 1. Taurine and hypotaurine metabolism, 2. Phenylalanine metabolism, 3. Steroid biosynthesis, 4. Tyrosine metabolism, 5. Glycerophospholipid metabolism, 6. Phenylalanine, tyrosine and tryptophan biosynthesis, 7. Sphingolipid metabolism, 8. beta-Alanine metabolism.

Based on the above results, HG showed better effects than LG in ameliorating NAFLD. Thus, fecal metabolomics was applied to compare metabolic profile changes among NG, MG, and HG. PCA score plots in positive and negative mode illustrated that high-fat diet significantly changed the metabolites of rat feces (Figures 3A,B). A separation of PCA between HG and MG was clearly shown in positive mode (Figure 3A). In negative mode, points from HG were also offset to some extent compared to MG (Figure 3B).

OPLS-DA models were established for pairwise comparison between groups and further differential metabolite identification. In OPLS-DA score plots, MG could separate with NG (Figures 3C,D) and HG (Figures 3G,H). The permutation test was used to evaluate the robustness of OPLS-DA model, the R^2^Y and Q^2^ values (Figures 3E,F,I,J) ensured no overfitting when modeling. OPLS-DA provided metabolites with VIP values greater than 1. Combining with student’s t-test (*p* < 0.05, the data was met normally distributed), the visual results of differential metabolites were exhibited as volcano plots (Figure 3K, L), and the specific metabolite information was listed in Table 1.

**TABLE1:** Identification of potential biomarkers of rat fecal samples.

Ion Mode	Name	Formula	RT(s)	Experimental mass	Actual mass	VIP	Relative content
[M + NH_4_]+	15-KETE	C_20_H_30_O_3_	4.36	336.2526	318.4504	1.39	NG<MG>HG
[M + H]+	2-Keto-6-acetamidocaproate	C_8_H_13_NO_4_	1.45	188.0917	187.1931	1.29	NG<MG>HG
[M + H]+	4a-Carboxy-4b-methyl-5a-cholesta-8,24-dien-3b-ol	C_29_H_46_O_3_	6.18	443.3473	442.6737	1.33	NG<MG>HG
[M + H]+	5-Amino-3-oxohexanoate	C_6_H_11_NO_3_	2.12	146.0811	145.1564	1.54	NG<MG>HG
[M + H]+	5-Methoxytryptophan	C_12_H_14_N_2_O_3_	6.22	235.1076	234.2512	1.48	NG<MG<HG
[M + H]+	6-Hydroxydopamine	C_8_H_11_NO_3_	3.86	170.0811	169.1778	1.54	NG<MG>HG
[M + Na]+	6-Keto-prostaglandin F1a	C_20_H_34_O_6_	7.68	393.2242	370.4804	1.10	NG<MG<HG
[M-H]-	8,9-DiHETr	C_20_H_34_O_4_	0.88	339.2525	338.4816	1.09	NG<MG>HG
[M + H]+	9-HOTE	C_18_H_30_O_3_	0.63	295.2263	294.4290	1.24	NG<MG>HG
[M-H]-	Acetylcysteine	C_5_H_9_NO_3_S	2.14	162.0225	163.1950	1.42	NG<MG>HG
[M + H]+	Benzaldehyde	C_7_H_6_O	4.06	107.0494	106.1219	1.40	NG<MG<HG
[M + H]+	Cholesta-4,6-dien-3-one	C_27_H_42_O	0.55	383.3300	382.6218	1.37	NG<MG>HG
[M + Na]+	DG(16:0/16:0/0:0)	C_35_H_68_O_5_	0.53	591.4984	568.9114	1.10	NG<MG>HG
[M + H]+	Dopamine	C_8_H_11_NO_2_	5.24	154.0862	153.1784	1.53	NG<MG>HG
[M + H]+	Dopamine glucuronide	C_14_H_19_NO_8_	7.50	330.1180	329.3026	1.51	NG<MG<HG
[M + H]+	gamma-Glutamylvaline	C_10_H_18_N_2_O_5_	6.54	247.1284	246.2630	1.30	NG<MG>HG
[M + H]+	Glycylprolylhydroxyproline	C_12_H_19_N_3_O_5_	6.70	286.1280	285.3000	1.05	NG<MG>HG
[M + H]+	Hexanoylglycine	C_8_H_15_NO_3_	5.43	174.1124	173.2096	1.04	NG<MG>HG
[M + H]+	Hippuric acid	C_9_H_9_NO_3_	6.10	180.0655	179.1727	1.16	NG<MG<HG
[M + H]+	Isoleucyl-Tyrosine	C_15_H_22_N_2_O_4_	6.78	295.1644	294.3462	1.30	NG<MG<HG
[M-H]-	Isolithocholic acid	C_24_H_40_O_3_	1.15	375.2902	376.5726	1.36	NG<MG>HG
[M]+	L-Acetylcarnitine	C_9_H_18_NO_4_	6.01	204.1230	204.2435	1.48	NG<MG>HG
[M-H]-	Lithocholic acid	C_24_H_40_O_3_	0.88	375.2903	376.5726	1.34	NG<MG>HG
[M + H]+	L-Kynurenine	C_10_H_12_N_2_O_3_	5.77	209.0921	208.2139	1.06	NG<MG>HG
[M + H]+	LysoPA(18:0e/0:0)	C_21_H_45_O_6_P	4.43	425.3002	424.5590	1.55	NG<MG>HG
[M + H]+	LysoPC(14:1(9Z))	C_22_H_44_NO_7_P	5.08	466.2905	465.5610	1.47	NG<MG>HG
[M + H]+	LysoPC(18:3(6Z,9Z,12Z))	C_26_H_48_NO_7_P	3.27	518.3171	517.6356	1.30	NG>MG<HG
[M + H]+	Methyldopa	C_10_H_13_NO_4_	6.53	212.0916	211.2145	1.51	NG<MG>HG
[M + H]+	Methylmalonic acid semialdehyde	C_4_H_6_O_3_	6.21	103.0393	102.0886	1.13	NG<MG<HG
[M + H]+	N-Acetyldopamine	C_10_H_13_NO_3_	4.55	196.0969	195.2151	1.45	NG<MG>HG
[M + H]+	N-Arachidonoyl glycine	C_22_H_35_NO_3_	4.19	362.2682	361.5182	1.41	NG<MG>HG
[M + H]+	Norepinephrine	C_8_H_11_NO_3_	4.45	170.0811	169.1778	1.03	NG<MG>HG
[M + H]+	PA(22:2(13Z,16Z)/16:0)	C_41_H_77_O_8_P	2.97	729.5441	729.0330	1.54	NG<MG>HG
[M + H]+	PE(24:1(15Z)/18:4(6Z,9Z,12Z,15Z))	C_47_H_84_NO_8_P	3.24	822.5863	822.1455	1.58	NG<MG>HG
[M + H]+	Phenylalanyl-Alanine	C_12_H_16_N_2_O_3_	6.60	237.1231	236.2670	1.06	NG<MG<HG
[M + H]+	Pivaloylcarnitine	C_12_H_23_NO_4_	5.08	246.1697	245.3153	1.39	NG<MG>HG
[M + H]+	Presqualene diphosphate	C_30_H_52_O_7_P_2_	2.31	587.3268	586.6772	1.27	NG<MG<HG
[M + H]+	PS(22:0/15:0)	C_43_H_84_NO_10_P	2.89	806.5915	806.1160	1.10	NG<MG>HG
[M + H]+	Pyridoxamine	C_8_H_12_N_2_O_2_	4.21	169.0970	168.1931	1.42	NG<MG<HG
[M + H]+	Saccharopine	C_11_H_20_N_2_O_6_	7.56	277.1390	276.2863	1.47	NG<MG>HG
[M + H]+	Serotonin	C_10_H_12_N_2_O	2.36	177.1022	176.2151	1.43	NG<MG>HG
[M + H]+	Threoninyl-Leucine	C_10_H_20_N_2_O_4_	6.51	233.1494	232.2800	1.47	NG<MG>HG
[M + H]+	Traumatic acid	C_12_H_20_O_4_	1.16	229.1433	228.2848	1.27	NG<MG>HG
[M-H]-	Tyramine	C_8_H_11_NO	4.05	136.0762	137.1790	1.20	NG<MG<HG
[M + H]+	Tyrosyl-Valine	C_14_H_20_N_2_O_4_	6.67	281.1492	280.3196	1.42	NG<MG<HG
[M + H]+	Ursodeoxycholic acid 3-sulfate	C_24_H_40_O_7_S	4.70	473.2584	472.6350	1.34	NG>MG<HG
[M + H]+	Valyl-Phenylalanine	C_14_H_20_N_2_O_3_	4.81	265.1541	264.3250	1.21	NG<MG<HG
[M + H]+	Vitamin D3	C_27_H_44_O	0.55	385.3457	384.6377	1.47	NG<MG>HG
[M + Na]+	xi-3-Hydroxy-5-phenylpentanoic acid O-beta-D-Glucopyranoside	C_17_H_24_O_8_	4.66	379.1357	356.3677	1.27	NG>MG<HG
[M-H]-	1,5-Anhydrosorbitol	C_6_H_12_O_5_	5.20	163.0608	164.1565	1.48	MG>NG
[M-H2O + H]+	13-L-Hydroperoxylinoleic acid	C_18_H_32_O_4_	0.85	295.2262	312.4443	1.05	MG>NG
[M-H]-	2,3-Dinor-TXB2	C_18_H_30_O_6_	5.42	341.1969	342.4272	1.25	MG>NG
[M-H]-	20-Carboxy-leukotriene B4	C_20_H_30_O_6_	5.92	365.1969	366.4486	1.07	MG<NG
[M-H]-	20-Hydroxy-PGF2a	C_20_H_34_O_6_	3.68	369.2283	370.4804	1.26	MG>NG
[M + H]+	3-Dehydrosphinganine	C_18_H_37_NO_2_	2.16	300.2891	299.4919	1.33	MG>NG
[M-H]-	3-Hydroxybenzoic acid	C_7_H_6_O_3_	3.21	137.0238	138.1220	1.41	MG<NG
[M-H]-	3-Methyladipic acid	C_7_H_12_O_4_	3.45	159.0658	160.1678	1.06	MG<NG
[M + H]+	3-Methyldioxyindole	C_9_H_9_NO_2_	0.65	164.0706	163.1733	1.06	MG<NG
[M-H]-	4-Hydroxyphenylpyruvic acid	C_9_H_8_O_4_	0.80	179.0346	180.1574	1.29	MG>NG
[M + H]+	5-HEPE	C_20_H_30_O_3_	2.41	301.2157	318.4504	1.31	MG>NG
[M + H]+	5-Methyldeoxycytidine	C_10_H_15_N_3_O_4_	5.75	242.1134	241.2438	1.19	MG<NG
[M + H]+	6,15-Diketo,13,14-dihydro-PGF1a	C_20_H_32_O_6_	5.45	369.2242	368.4645	1.22	MG>NG
[M-H]-	8-iso-15-keto-PGE2	C_20_H_30_O_5_	4.02	349.2021	350.4492	1.14	MG<NG
[M + H]+	Adenine	C_5_H_5_N_5_	2.88	136.0617	135.1267	1.16	MG<NG
[M-H]-			2.86	134.0466		1.28	
[M + H]+	Alanyl-Proline	C_8_H_14_N_2_O_3_	5.80	187.1078	186.2110	1.02	MG<NG
[M + H]+	Aminoadipic acid	C_6_H_11_NO_4_	7.79	162.0761	161.1558	1.01	MG>NG
[M + H]+	Anserine	C_10_H_16_N_4_O_3_	7.26	241.1295	240.2590	1.38	MG>NG
[M-H]-			7.27	239.1150		1.49	
[M-H]-	Arachidonic acid	C_20_H_32_O_2_	0.73	303.2330	304.4669	1.08	MG>NG
[M-H]-	Ascorbic acid	C_6_H_8_O_6_	0.91	175.0244	176.1241	1.34	MG>NG
[M + H]+	Biliverdin	C_33_H_34_N_4_O_6_	4.36	583.2541	582.6570	1.06	MG>NG
[M + Na]+	Cellobiose	C_12_H_22_O_11_	6.22	365.1046	342.2965	1.56	MG>NG
[M-H2O + H]+	Cholesterol	C_27_H_46_O	0.53	369.3509	386.6535	1.34	MG>NG
[M + H]+	CPA(16:0/0:0)	C_19_H_37_O_6_P	5.96	393.2378	392.4672	1.44	MG>NG
[M + H]+	Deoxycytidine	C_9_H_13_N_3_O_4_	2.13	228.0976	227.2172	1.05	MG>NG
[M-H]-	Deoxyinosine	C_10_H_12_N_4_O_4_	3.28	251.0784	252.2300	1.08	MG<NG
[M + H]+	Dihydrothymine	C_5_H_8_N_2_O_2_	4.22	129.0660	128.1310	1.13	MG<NG
[M + H]+	Dopamine 3-O-sulfate	C_8_H_11_NO_5_S	0.78	234.0429	233.2420	1.24	MG<NG
[M + H]+	Eicosapentaenoic acid	C_20_H_30_O_2_	0.91	303.2312	302.4510	1.02	MG>NG
[M + H]+	Ercalcitriol	C_28_H_44_O_3_	2.74	411.3290	428.6472	1.11	MG>NG
[M + H]+	gamma-Glutamylleucine	C_11_H_20_N_2_O_5_	7.21	261.1441	260.2869	1.14	MG>NG
[M-H]-	Glyceraldehyde	C_3_H_6_O_3_	2.58	89.0236	90.0779	1.32	MG<NG
[M + H]+	Glycerophosphocholine	C_8_H_20_NO_6_P	2.58	258.1082	257.2230	1.32	MG>NG
[M-H]-	Glycocholic acid	C_26_H_43_NO_6_	4.27	464.3015	465.6227	1.51	MG>NG
[M + H]+	Glycoursodeoxycholic acid	C_26_H_43_NO_5_	4.21	450.3208	449.6233	1.04	MG>NG
[M-H]-	Guanine	C_5_H_5_N_5_O	4.17	150.0417	151.1261	1.07	MG<NG
[M-H]-	Hexadecanedioic acid	C_16_H_30_O_4_	3.42	285.2069	286.4070	1.46	MG<NG
[M + H]+	Histidinal	C_6_H_9_N_3_O	1.67	140.0818	139.1580	1.21	MG<NG
[M + H]+	Homocitrulline	C_7_H_15_N_3_O_3_	6.37	190.1185	189.2123	1.00	MG<NG
[M + H]+	Hydroxyphenylacetylglycine	C_10_H_11_NO_4_	4.45	210.0760	209.1986	1.27	MG>NG
[M-H]-	Hypogeic acid	C_16_H_30_O_2_	0.76	253.2171	254.4082	1.40	MG>NG
[M + H]+	Hypoxanthine	C_5_H_4_N_4_O	3.07	137.0457	136.1115	1.13	MG<NG
[M-H]-			3.05	135.0306		1.14	
[M + H]+	Imidazoleacetic acid	C_5_H_6_N_2_O_2_	5.70	127.0503	126.1133	1.22	MG<NG
[M-H]-	Lipoxin A4	C_20_H_32_O_5_	1.39	351.2177	352.4651	1.04	MG<NG
[M + H]+	L-Palmitoylcarnitine	C_23_H_45_NO_4_	3.40	400.3417	399.6160	1.44	MG>NG
[M + H]+	Lutein	C_40_H_56_O_2_	0.55	568.4265	568.8860	1.51	MG<NG
[M + H]+	LysoPE(0:0/20:5(5Z,8Z,11Z,14Z,17Z))	C_25_H_42_NO_7_P	7.50	500.2713	499.5772	1.54	MG>NG
[M + H]+	LysoPE(15:0/0:0)	C_20_H_42_NO_7_P	3.82	440.2763	439.5237	1.36	MG<NG
[M + H]+	MG(24:6(6Z,9Z,12Z,15Z,18Z,21Z)/0:0/0:0)	C_27_H_42_O_4_	4.10	431.3147	430.6200	1.44	MG>NG
[M-H]-	myo-Inositol	C_6_H_12_O_6_	5.31	179.0557	180.1559	1.38	MG>NG
[M + H]+	N-Acetylglutamine	C_7_H_12_N_2_O_4_	5.48	189.0869	188.1812	1.13	MG<NG
[M + H]+	N-Acetyl-L-tyrosine	C_11_H_13_NO_4_	5.35	224.0915	223.2252	1.15	MG<NG
[M + H]+	Norvaline	C_5_H_11_NO_2_	0.30	118.0865	117.1463	1.10	MG<NG
[M + H]+	N-Succinyl-L,L-2,6-diaminopimelate	C_11_H_18_N_2_O_7_	7.05	291.1182	290.2698	1.11	MG>NG
[M + H]+	Oleamide	C_18_H_35_NO	0.97	282.2787	281.4766	1.53	MG>NG
[M-H]-	Oleoyl glycine	C_20_H_37_NO_3_	1.74	338.2700	339.5127	1.23	MG>NG
[M-H]-	Orotic acid	C_5_H_4_N_2_O_4_	1.28	157.0138	156.0963	1.06	MG>NG
[M + H]+	Palmitoleoyl Ethanolamide	C_18_H_35_NO_2_	1.42	298.2735	297.4760	1.30	MG>NG
[M-H]-	Pelargonic acid	C_9_H_18_O_2_	0.88	157.1229	158.2380	1.06	MG<NG
[M-H]-	Phenylacetylglycine	C_10_H_11_NO_3_	3.17	192.0663	193.1992	1.09	MG<NG
[M-H]-	Pimelic acid	C_7_H_12_O_4_	0.18	159.0657	160.1678	1.09	MG<NG
[M + H]+	Pipecolic acid	C_6_H_11_NO_2_	8.63	130.0863	129.1570	1.18	MG>NG
[M + H]+	Porphobilinogen	C_10_H_14_N_2_O_4_	1.92	227.1025	226.2292	1.04	MG<NG
[M + H]+	Prolylhydroxyproline	C_10_H_16_N_2_O_4_	7.69	229.1181	228.2450	1.16	MG>NG
[M-H]-	Prostaglandin A2	C_20_H_30_O_4_	1.76	333.2073	334.4498	1.28	MG<NG
[M-H]-	Prostaglandin B1	C_20_H_32_O_4_	1.70	335.2227	336.4657	1.39	MG<NG
[M-H]-	Prostaglandin E3	C_20_H_30_O_5_	5.54	349.2020	350.4492	1.54	MG<NG
[M-H]-	Prostaglandin G2	C_20_H_32_O_6_	4.63	367.2129	368.4645	1.07	MG>NG
[M-H]-	Pyroglutamic acid	C_5_H_7_NO_3_	5.53	128.0347	129.1140	1.36	MG<NG
[M + H]+	S-(2-Methylpropionyl)-dihydrolipoamide-E	C_12_H_23_NO_2_S_2_	6.83	278.1230	277.4470	1.20	MG>NG
[M-H]-	Sebacic acid	C_10_H_18_O_4_	0.97	201.1129	202.2475	1.11	MG>NG
[M-H]-	Shikimic acid	C_7_H_10_O_5_	2.70	173.0452	174.1513	1.06	MG<NG
[M + H]+	SM(d18:1/16:0)	C_39_H_79_N_2_O_6_P	3.48	703.5731	703.0281	1.47	MG>NG
[M + H]+	Sphingosine	C_18_H_37_NO_2_	1.50	300.2890	299.4919	1.51	MG>NG
[M + H]+	Stearidonic acid	C_18_H_28_O_2_	3.63	277.2157	276.4137	1.26	MG>NG
[M-H]-	Suberic acid	C_8_H_14_O_4_	5.20	173.0815	174.1944	1.15	MG<NG
[M-H]-	Sucrose	C_12_H_22_O_11_	7.39	341.1089	342.2965	1.45	MG>NG
[M + H]+	Taurine	C_2_H_7_NO_3_S	5.27	126.0220	125.1470	1.24	MG<NG
[M-H]-			5.26	124.0068		1.28	
[M + H]+	Threoninyl-Phenylalanine	C_13_H_18_N_2_O_4_	1.99	267.1335	266.2970	1.55	MG<NG
[M-H]-	Thromboxane B3	C_20_H_32_O_6_	3.39	367.2129	368.4645	1.40	MG<NG
[M-H]-	Thymidine	C_10_H_14_N_2_O_5_	1.52	241.0827	242.2286	1.15	MG<NG
[M + H]+	Tiglylglycine	C_7_H_11_NO_3_	7.12	158.0813	157.1671	1.52	MG<NG
[M + H]+	Trigonelline	C_7_H_7_NO_2_	2.71	138.0549	137.1360	1.16	MG<NG
[M-H]-	Uridine 5′-monophosphate	C_9_H_13_N_2_O_9_P	7.63	323.0285	324.1813	1.05	MG>NG
[M-H2O + H]+	Ursodeoxycholic acid	C_24_H_40_O_4_	2.73	375.2883	392.5720	1.12	MG>NG
[M-H2O + H]+	Ursolic acid	C_30_H_48_O_3_	0.61	439.3564	456.7110	1.16	MG<NG
[M + H]+	(R)-Salsolinol	C_10_H_13_NO_2_	3.96	180.1019	179.2157	1.52	HG<MG
[M + H]+	11-Dehydro-thromboxane B2	C_20_H_32_O_6_	4.07	369.2240	368.4645	1.43	HG<MG
[M + H]+	2-Phenylacetamide	C_8_H_9_NO	4.07	136.0757	135.1632	1.53	HG<MG
[M + H]+	2-Piperidinone	C_5_H_9_NO	4.70	100.0760	99.1311	1.24	HG<MG
[M-H]-	8-Isoprostaglandin E1	C_20_H_34_O_5_	2.71	353.2332	354.4810	1.81	HG<MG
[M + H]+	Androstenedione	C_19_H_26_O_2_	1.04	287.2001	286.4085	1.23	HG>MG
[M + H]+	beta-Alanine	C_3_H_7_NO_2_	0.88	90.0554	89.0932	1.14	HG<MG
[M + H]+	Biotin	C_10_H_16_N_2_O_3_S	4.81	245.0951	244.3110	1.14	HG<MG
[M-H]-	Butyrylcarnitine	C_11_H_21_NO_4_	4.15	230.1476	231.2920	1.72	HG<MG
[M + H]+	Corticosterone	C_21_H_30_O_4_	2.95	347.2207	346.4605	1.21	HG<MG
[M + H]+	Hexadecanedioic acid mono-L-carnitine ester	C_23_H_43_NO_6_	5.68	430.3158	429.5906	1.24	HG<MG
[M + H]+	Isoleucyl-Serine	C_9_H_18_N_2_O_4_	4.77	219.1338	218.2502	1.12	HG<MG
[M + H]+	Leucyl-Tyrosine	C_15_H_22_N_2_O_4_	6.41	295.1648	294.3462	1.39	HG<MG
[M + H]+	L-Histidine	C_6_H_9_N_3_O_2_	4.05	156.0767	155.1546	1.39	HG<MG
[M + H]+	L-Tyrosine	C_9_H_11_NO_3_	5.43	182.0812	181.1885	1.03	HG<MG
[M + H]+	Lysyl-Proline	C_11_H_21_N_3_O_3_	5.04	244.1651	243.3070	1.54	HG<MG
[M + H]+	N-a-Acetylcitrulline	C_8_H_15_N_3_O_4_	7.42	218.1135	217.2224	1.24	HG<MG
[M + H]+	N-Acetylleucine	C_8_H_15_NO_3_	5.67	174.1125	173.2096	1.38	HG<MG
[M + H]+	O-Phosphoethanolamine	C_2_H_8_NO_4_P	3.57	142.0264	141.0630	1.15	HG<MG
[M + H]+	Phenylalanyl-Valine	C_14_H_20_N_2_O_3_	5.54	265.1542	264.3250	1.40	HG<MG
[M + H]+	Prolylphenylalanine	C_14_H_18_N_2_O_3_	4.31	263.1386	262.3090	1.10	HG<MG
[M + Na]+	Prostaglandin D3	C_20_H_30_O_5_	7.46	373.1963	350.4490	1.10	HG<MG
[M + H]+	Prostaglandin I2	C_20_H_32_O_5_	3.05	353.2315	352.4651	1.26	HG<MG
[M-H]-	Ribothymidine	C_10_H_14_N_2_O_6_	2.60	257.0779	258.2280	2.34	HG<MG
[M + H]+	Sphinganine	C_18_H_39_NO_2_	2.29	302.3048	301.5078	1.37	HG<MG
[M + H]+	Taurocholic acid	C_26_H_45_NO_7_S	1.36	516.3010	515.7030	1.67	HG<MG
[M-H]-	Tetradecanedioic acid	C_14_H_26_O_4_	4.04	257.1758	258.3538	1.74	HG<MG
[M + H]+	Trimethylaminoacetone	C_6_H_14_NO	5.04	116.1072	116.1815	1.10	HG<MG
[M + H]+	Valyl-Valine	C_10_H_20_N_2_O_3_	3.82	217.1545	216.2810	1.04	HG<MG
[M-H2O + H]+	Vitamin A	C_20_H_30_O	0.55	269.2257	286.4516	1.05	HG<MG

### 3.4 Analysis of Content Change and Biological Significance of Typical Biomarkers

This study detected 134 differential metabolites between NG and MG, 80 differential metabolites between MG and HG. Metabolites found in NG and MG indicated the impact of NAFLD, while metabolites found in MG and HG suggested the role of diosgenin in NAFLD. Some representative metabolites were selected, and their MS/MS spectrums and structural information of fragment ions were shown in Supplementary Figure S2. NAFLD primarily altered lysine degradation, linoleic acid metabolism, taurine and hypotaurine metabolism, sphingolipid metabolism, glycerophospholipid metabolism, arachidonic acid metabolism, steroid biosynthesis, tyrosine metabolism, as well as ubiquinone and other terpenoid-quinone biosynthesis of rats (Figure 3M). And diosgenin primarily altered taurine and hypotaurine metabolism, phenylalanine metabolism, steroid biosynthesis, tyrosine metabolism, glycerophospholipid metabolism, phenylalanine, tyrosine, and tryptophan biosynthesis, sphingolipid metabolism, and beta-alanine metabolism of NAFLD rats (Figure 3N).

**FIGURE 4:**
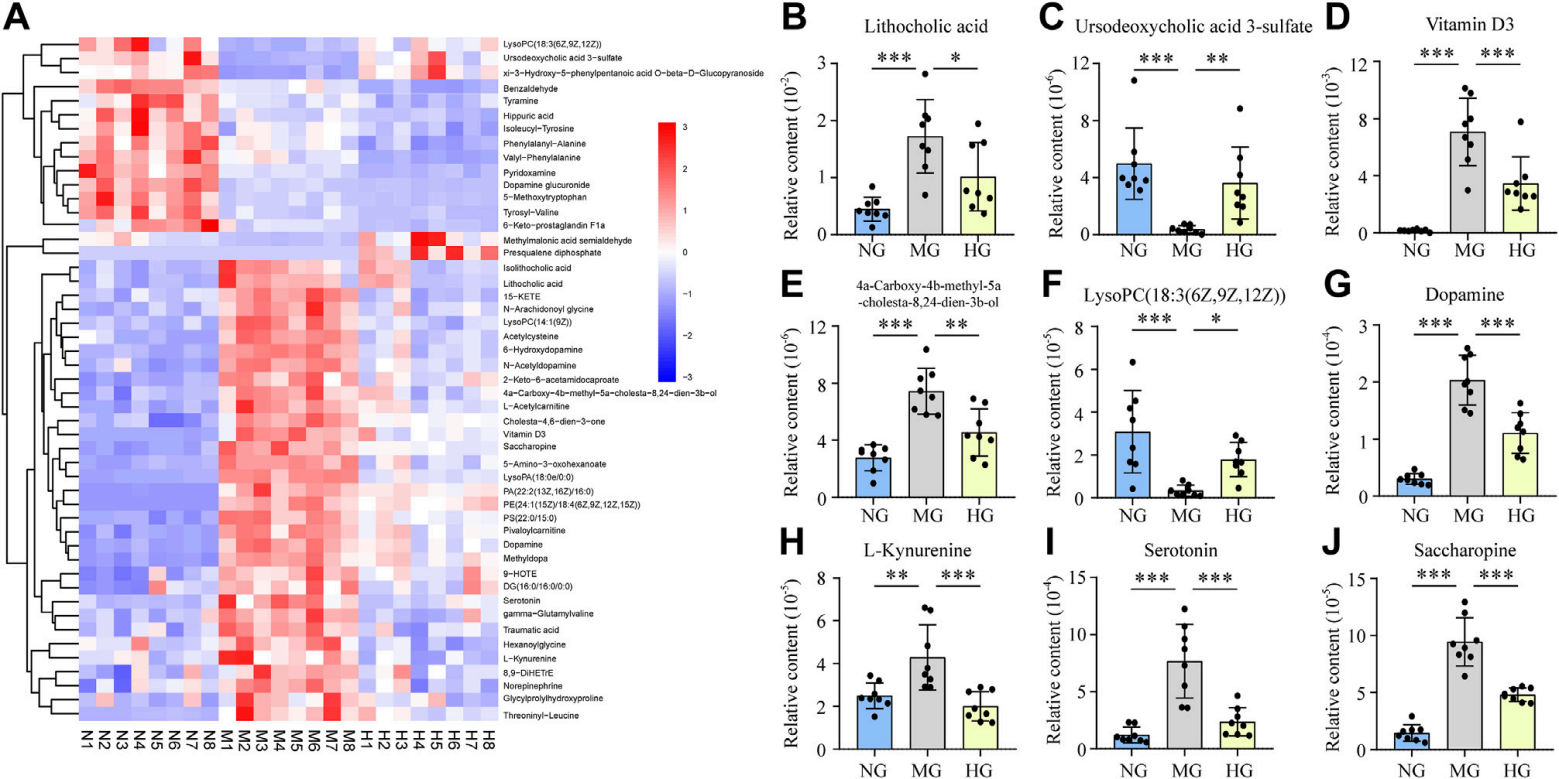
Relative contents of potential biomarkers in NG, MG, and HG, ***p < 0.001, **p < 0.01, *p < 0.05 **(A)** heatmap expressed relative contents of potential biomarkers, histograms expressed relative contents of lithocholic acid **(B)**, ursodeoxycholic acid 3-sulfate **(C)**, vitamin D3 **(D)**, 4a-carboxy-4b-methyl-5a-cholesta-8,24-dien-3b-ol **(E)**, lysoPC(18:3(6Z,9Z,12Z)) **(F)**, dopamine **(G)**, L-kynurenine **(H)**, serotonin **(I)**, and saccharopine **(J)**.

Dramatically, totally 49 of these metabolites were discovered repeatedly, which indicated that both NAFLD and diosgenin changed their contents distinctly. The content of these metabolites in each sample was exhibited as a heatmap (Figure 4A). Among these 49 metabolites, diosgenin reversed content changes of 36 metabolites induced by NAFLD.

#### 3.4.1 Lipid Metabolism

Lipid metabolism disorder is a typical characteristic of NAFLD, and we exactly discovered many biomarkers (NG vs. MG) classified as lipids and lipid-like molecules. And diosgenin exhibited observable effects on many of these lipids and lipid-like molecules.

Lithocholic acid (LCA) is a secondary bile acid formed from chenodeoxycholate by bacterial 7-dehydroxylation. The current study found that LCA from NAFLD rats was much higher than normal rats, while diosgenin decreased LCA markedly (Figure 4B). Because the content of chenodesoxycholic acid or chenodeoxycholate was not found any difference, 7-dehydroxylation regulated by gut microbiota might play a crucial role in diosgenin led LCA down-regulation (Jiao et al., 2018). Isolithocholic acid is another product of chenodeoxycholate by bacterial action. Literature about isolithocholic acid is much less than LAC. One report has found higher levels of LAC and isolithocholic acid in fecal samples of diet-induced obese mice (de Groot et al., 2020). Our study found the same phenomenon, and diosgenin could down-regulate the level of isolithocholic acid (Figure 4A). Ursodeoxycholic acid 3-sulfate is the 3-sulfate conjugate of Ursodeoxycholic acid (UDCA) (Nadinskaia et al., 2021). Out results illuminated a down-regulation of ursodeoxycholic acid 3-sulfate in high-fat diet induced NAFLD rats, and diosgenin significantly mitigated this down-regulation (Figure 4C). Additionally, MG significantly increased the levels of glycocholic acid, glycoursodeoxycholic acid, and UDCA, while diosgenin could not change these three bile acids compared to MG. Diosgenin down-regulated taurocholic acid, which has been reported to be increased in NAFLD mice (Xiang Zhang et al., 2021).

Vitamin D3, also termed cholecalciferol is a steroid hormone predominantly synthesized in the liver and involved in steroid biosynthesis. However, Vitamin D3 in feces from NAFLD patients or animals has not been reported. Our results demonstrated a surprising increase in Vitamin D3 in NAFLD rats. Compared to MG, the Vitamin D3 in HG was significantly lower (Figure 4D). 4a-Carboxy-4b-methyl-5a-cholesta-8,24-dien-3b-ol is an intermediate in cholesterol biosynthesis and cholesta-4,6-dien-3-one is a derivative of cholesterol. These two cholesterol-related biomarkers increased in MG and were down-regulated by diosgenin (Figures 4A,E). We indeed detected a higher cholesterol level in MG compared to NG. In contrast, the effect of diosgenin on cholesterol was not observed by fecal metabolomics analysis.

15-KETE, 6-Keto-prostaglandin F1a, and 8,9-DiHETrE belonged to class fatty acyls that participated in arachidonic acid metabolism and were all up-regulated in MG. 6-Keto-prostaglandin F1a was reported to show a significant positive correlation with the level of high density lipoprotein cholesterol in plasma (Symons, 1990). Diosgenin decreased the level of 5-KETE and 6-Keto-prostaglandin F1a, while increasing the level of 8,9-DiHETrE. Hexanoylglycine and L-acetylcarnitine are two lipids involved in fatty acid oxidation. The current study discovered a higher level of these two biomarkers in NAFLD rats, and diosgenin inhibited the increase. Traumatic acid participates in alpha-linolenic acid metabolism, which high level in NAFLD rats was also significantly decreased by diosgenin.

An enormous amount of glycerophospholipids showed changes in content after high-fat diet fed. And diosgenin exerted an observable effect on glycerophospholipids. In total, six glycerophospholipids were regulated by diosgenin. LysoPA(18:0e/0:0), LysoPC(14:1(9Z)), PA(22:2(13Z,16Z)/16:0), PE(24:1(15Z)/18:4(6Z,9Z,12Z,15Z)), and PS(22:0/15:0) were increased in MG, while HG exhibited a dramatically down-regulation. Interestingly, LysoPC(18:3(6Z,9Z,12Z)) showed an opposite trend with other glycerophospholipids. Its relative content in NG and HG was high, while very low in MG (Figure 4F). Therefore, the effect of diosgenin on glycerophospholipids was not simply a downward adjustment but a precise regulation. Additionally, diosgenin inhibited the increase of DG(16:0/16:0/0:0), a glycerolipid involved in phospholipid biosynthesis and glycerolipid metabolism.

#### 3.4.2 Amino Acid Metabolism

As described above, amino acid metabolism disorder was another typical characteristic of NAFLD patients. This study also found some biomarkers related to amino acid metabolism, especially AAAs metabolism.

Dopamine, norepinephrine, and tyramine are all metabolites of tyrosine. These two common compounds are closely associated with nervous system diseases like Alzheimer’s disease and Parkinson’s disease (Tang et al., 2018). Nevertheless, the study about their functions or relationship with NAFLD was inadequate. Thus, the current study regarded them as common biomarkers in tyrosine metabolism. Dopamine, norepinephrine, and tyramine were all up-regulated in the fecal samples of NAFLD rats. And diosgenin treatment displayed different effects on them. Dopamine and norepinephrine were deservedly decreased in HG, while tyramine was further up-regulated (Figures 4A,G). In addition, three kinds of dopamine derivatives/metabolites, dopamine glucuronide, N-acetyldopamine, dopamine 3-O-sulfate were equally deserving of attention. Dopamine glucuronide is generated in the liver by UDP glucuonyltransferase catalytic reaction, and dopamine is the substrate. Its content was higher in MG than NG and highest in HG among the three groups. N-acetyldopamine is an acetylated form of dopamine, whose high level in NAFLD rats was down-regulated by diosgenin. Dopamine 3-O-sulfate, a sulfonated form of dopamine, was observed content difference. Nevertheless, dopamine 3-O-sulfate was decreased in MG, and no significant difference was discovered between MG and HG.

L-Kynurenine is a central compound of the tryptophan metabolism pathway. This current study suggested L-Kynurenine was increased in NAFLD rats (Figure 4H), which was similar to the carbon tetrachloride induced liver injury rats (Liu et al., 2019). Distinctly, diosgenin inhibited the L-Kynurenine up-regulation in NAFLD rats (Figure 4H). Our study also discovered a high level of serotonin (also named 5-hydroxytryptamine, a neurotransmitter synthesized from tryptophan) in fecal samples of NAFLD rats, which was reduced by diosgenin significantly (Figure 4I). Hippuric acid, a compound related to phenylalanine metabolism, is a biomarker of various diseases such as obesity (Cho et al., 2017). Hippuric acid is formed by benzoic acid in the liver and is regarded as an index to evaluate liver function (Akira et al., 1997; Liu et al., 2019). This study discovered a high level of hippuric acid in MG. Regrettably, diosgenin further exacerbated the upward trend.

5-Amino-3-oxohexanoate is an intermediate in lysine degradation and saccharopine participates in lysine biosynthesis and degradation. These two lysine metabolism related biomarkers were high in NAFLD rats while decreased by diosgenin (Figures 4A,J). This result suggested diosgenin played a positive role in lysine metabolism.

### 3.5 Gut Microbiota Disorder in MG and Regulation in HG

**FIGURE 5:**
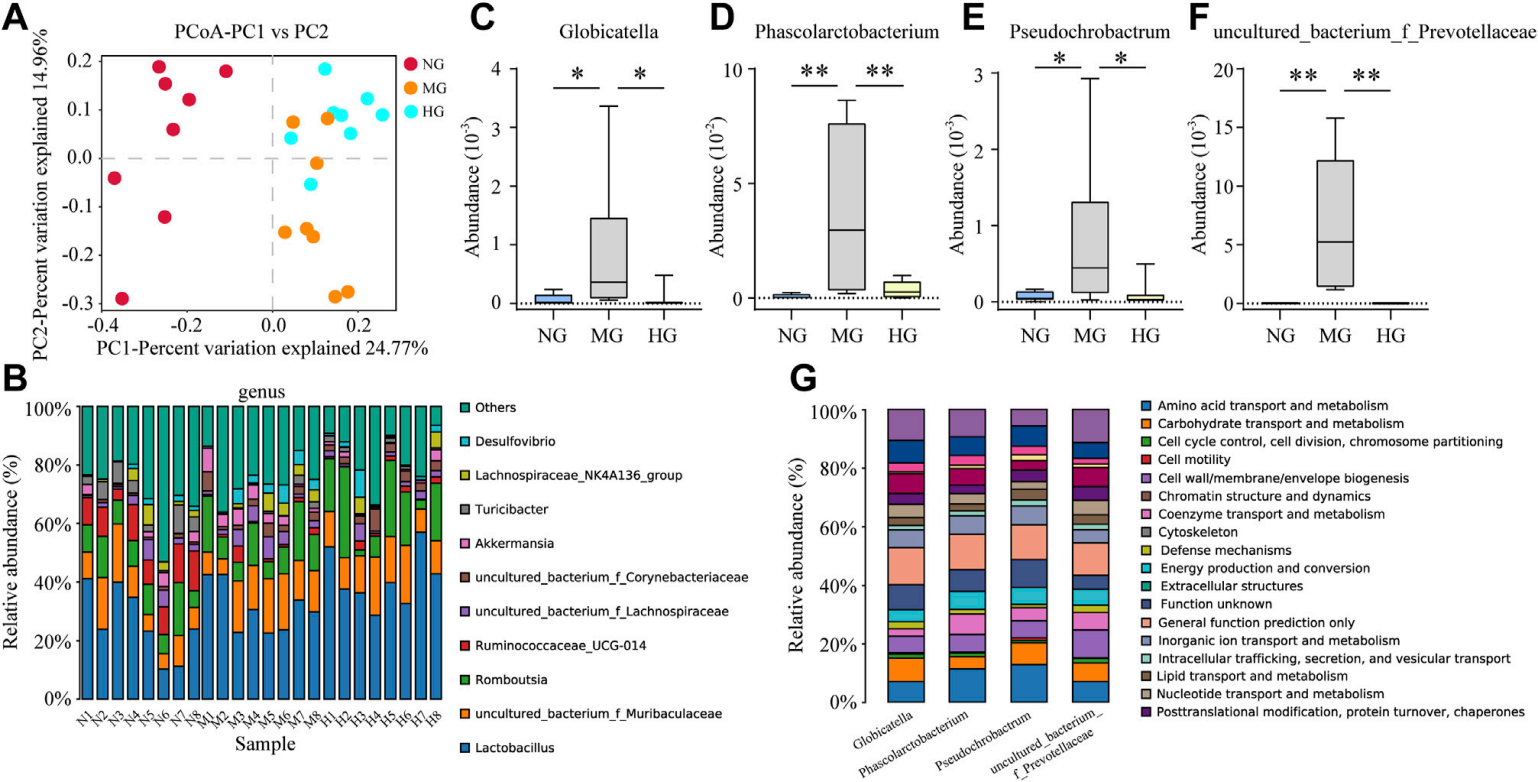
Gut microbiota analysis of NG, MG, and HG **(A)** PCoA score plots; **(B)** gut microbiota composition profile at genus level; abundance of Globicatella **(C)**, Phascolarctobacterium **(D)**, Pseudochrobactrum **(E)**, and uncultured_bacterium_f_Prevotellaceae **(F)**, **p < 0.01, *p < 0.05; **(G)** biological function prediction of Globicatella, Phascolarctobacterium, Pseudochrobactrum, and uncultured_bacterium_f_Prevotellaceae.

The principal coordinate analysis (PCoA) provides information about gut microbiota composition. NG and MG showed a significant separation (Figure 5A), which suggested noteworthy gut microbiota disorder in NAFLD rats. Though points of MG and HG in PCoA were not completely separated, a distinct tendency for separation was still visible (Figure 5A). Identification at levels of phylum, class, order, family, genus and species was analyzed in more detail (Figure 5B, Supplementary Figure S3A-E). At the genus level, *Globicatella, Phascolarctobacterium, Pseudochrobactrum, and uncultured_bacterium_f_*Prevotellaceae were increased in MG and down-regulated by diosgenin (Figures 5C–F). Thus, the functions of these four bacterial genera were closely concerned, and a database named COG (Clusters of Orthologous Groups of proteins) was adopted for function prediction. The prediction results indicated that amino acid/lipid transport and metabolism were involved in the functions of these bacterial genera (Figure 5G).

### 3.6 Relevance Analysis Between Biomarkers and Gut Microbiota

**FIGURE 6:**
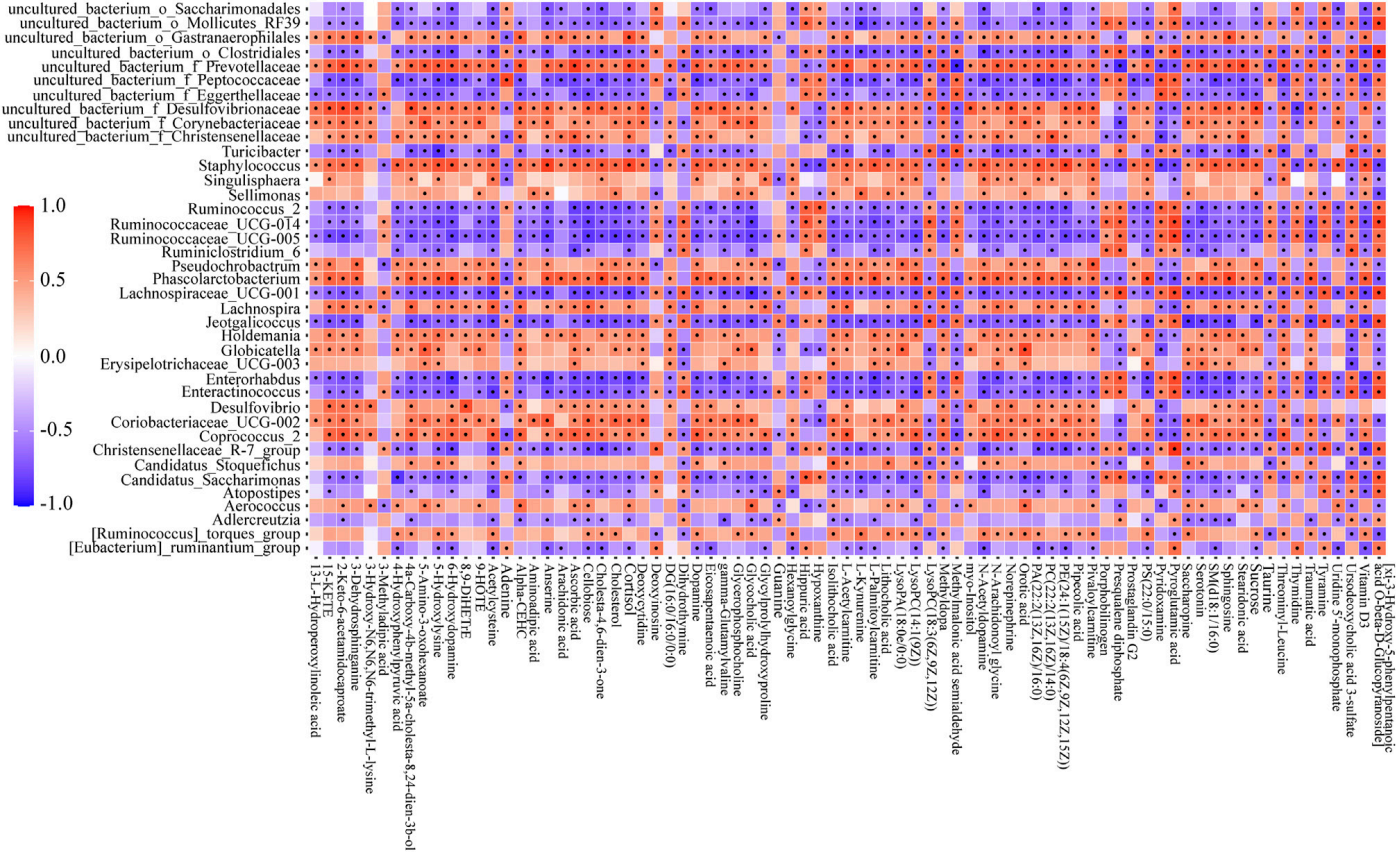
Correlation heatmap between perturbed gut microbiota genera and altered biomarkers in NG and MG. Significant correlations (r > 0.5 or r < −0.5, *p* < 0.05) were marked with dots.

Pearson’s correlation analysis was performed to study the correlation between biomarkers change and gut microbiota disorder. In consideration of too many biomarkers and bacterial genera were detected, 79 representative biomarkers (in specific metabolic pathways or closely associated with NAFLD) and 39 bacterial genera (largest abundance changes in MG) were selected as research objects. In the analysis, many biomarkers and bacterial genera had close connection (Figure 6, range for correlation, r > 0.5 or r < −0.5; *p* < 0.05). Taking lithocholic acid as example, *Candidatus_Stoquefichus, Coriobacteriaceae_UCG-002, Staphylococcus*, and other 13 bacterial genera were positively correlated with lithocholic acid, while *Lachnospiraceae_UCG-001, Ruminococcaceae_UCG-014, Turicibacter*, and other 13 bacterial genera showed negative correlations. To sum up, biomarkers change and gut microbiota disorder showed a strong connection in NAFLD rats. Consequently, the adjustment of diosgenin on gut microbiota might play a crucial role in regulating lipid/amino acid metabolism.

**FIGURE 7:**
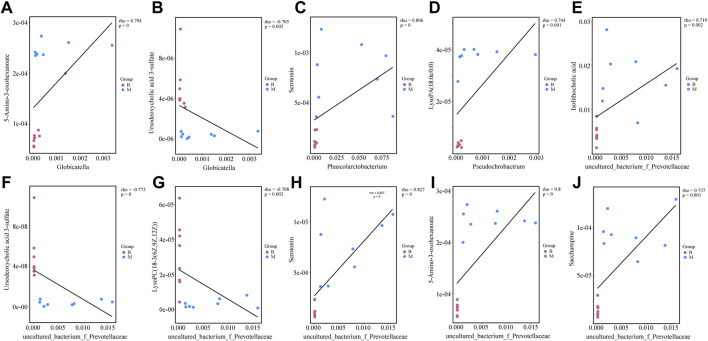
Correlations between gut microbiota genera and biomarkers regulated by diosgenin **(A)** correlation between *Globicatella* and 5-amino-3-oxohexanoate; **(B)** correlation between *Globicatella* and ursodeoxycholic acid 3-sulfate; **(C)** correlation between *Phascolarctobacterium* and serotonin; **(D)** correlation between *Pseudochrobactrum* and lysoPA(18:0e/0:0); **(E)** correlation between *uncultured_bacterium_f_Prevotellaceae* and isolithocholic acid; **(F)** correlation between *uncultured_bacterium_f_Prevotellaceae* and ursodeoxycholic acid 3-sulfate; **(G)** correlation between *uncultured_bacterium_f_Prevotellaceae* and lysoPC(18-3(6Z,9Z,12Z)); **(H)** correlation between *uncultured_bacterium_f_Prevotellaceae* and serotonin; **(I)** correlation between *uncultured_bacterium_f_Prevotellaceae* and 5-amino-3-oxohexanoate; **(J)** correlation between *uncultured_bacterium_f_Prevotellaceae* and saccharopine.

The relationships between four bacterial genera regulated by diosgenin and biomarkers emphasized the potential therapeutic effects of diosgenin on NAFLD. *Globicatella* was positively correlated with 38 biomarkers including 5-amino-3-oxohexanoate (Figure 7A, r = 0.794, *p* = 0) and negatively correlated with 10 biomarkers including ursodeoxycholic acid 3-sulfate (Figure 7B, r = -0.705, *p* = 0.002). Thus, *Globicatella* might involve in lysine and bile acid metabolism. *Phascolarctobacterium* was positively correlated with 58 biomarkers and negatively correlated with 14 biomarkers. Serotonin (Figure 7C, r = 0.806, *p* = 0) was an important biomarker, which suggested the effect of *Phascolarctobacterium* on tryptophan metabolism. *Pseudochrobactrum* was positively correlated with 42 biomarkers and negatively correlated with nine biomarkers. LysoPA(18:0e/0:0) (Figure 7D, r = 0.744, *p* = 0.001) showed a significant relevance to *Pseudochrobactrum*. Hence, it could be speculated that *Pseudochrobactrum* impacted glycerophospholipids metabolism. *Uncultured_bacterium_f_*Prevotellaceae was positively correlated with 58 biomarkers and negatively correlated with 15 biomarkers. Isolithocholic acid, ursodeoxycholic acid 3-sulfate, lysoPC(18-3(6Z,9Z,12Z)), serotonin, 5-amino-3-oxohexanoate, and saccharopine were all included when the r values were greater than 0.7 and *p* values were less than 0.01. These biomarkers were involved in lipid and amino acid metabolism as described above. To sum up, diosgenin restored the gut microbiota disorder and abnormal lipid/amino acid metabolism to a certain degree. Regulated bacterial genera and biomarkers exhibited a strong correlation, cooperatively ameliorated NAFLD.

## 4 Discussion

This study attempted to elucidate the mechanism of diosgenin in ameliorating NAFLD through gut microbiota regulation and related lipid/amino acid metabolism. We found many lipids, lipid-like molecules and amino acid metabolites. Diosgenin showed positive regulation of some bile acids with important biological significance, such as LCA, ursodeoxycholic acid 3-sulfate, and so on. The negative role LCA of in primary biliary cholangitis, nonalcoholic steatohepatitis and other liver diseases has already been recognized (King and Schoenfield, 1972; Funabashi et al., 2020). And diosgenin decreased this toxic bile acid, by which liver damage was expected to be alleviated. Ursodeoxycholic acid 3-sulfate is formed by UDCA, a bile acid applied for treating fatty liver and other liver diseases clinically by increasing bile acid secretion, regulating bile acid composition, and decreasing cholesterol (Nadinskaia et al., 2021). The abnormal decrease of ursodeoxycholic acid 3-sulfate in MG and the up-regulation effect of diosgenin were verified (Figure 4C). However, no significant difference of UDCA between MG and HG was detected in this experiment. Compared with NG, the UDCA level in MG was higher (Table 1). In other words, in the feces of normal rats, the UDCA tended to be sulfated while NAFLD rats showed the opposite trend. More importantly, diosgenin treatment could reverse the trend induced by NAFLD.

Another under-regulated biomarker, Vitamin D3, which low serum level and the correlation with NAFLD has been confirmed (Kitson and Roberts, 2012; Patel et al., 2016). Vitamin D3 intake was attempted to regulate the gut microbiota composition of cirrhotic rats (Lee et al., 2021) and gut microbiota in healthy volunteers (Bashir et al., 2016). These reports suggested Vitamin D3 also played a positive role in gut microbiota regulation. Nevertheless, no study on Vitamin D3 to regulate gut microbiota in NAFLD was reported. This current study discovered the effect of diosgenin on both Vitamin D3 and gut microbiota. And further investigation about their relevance in NAFLD was needed.

Hexanoylglycine and L-acetylcarnitine are two metabolites of fatty acid oxidation down-regulated by diosgenin, which was increased in elevated serum triglycerides related liver dysfunction mice (Li et al., 2013). L-Acetylcarnitine facilitates the movement of acetyl-CoA into the matrices of mitochondria during fatty acid oxidation (Li and Zhao, 2021). Also, being a food additive, L-acetylcarnitine was commonly used to lose weight because it promotes fatty acid oxidation. Recently, the function of L-acetylcarnitine in NAFLD also drew much attention (Li and Zhao, 2021). A serum metabolomic research found a significant increase of L-acetylcarnitine in NAFLD patients (Yang et al., 2021).

Serotonin is a biomarker in amino acid metabolic pathway worth discussing. Serotonin was widely reported in NAFLD research. For example, serotonergic system dysfunction in the intestine promoted bacterial endotoxin (LPS) to translocate into the liver, which could exacerbate NAFLD progression (Ke Zhang et al., 2020). And serotonin has already been regarded as a promising target in treating NAFLD since it plays a pivotal role in promoting liver fat synthesis and inhibiting fat degradation (Yabut et al., 2019). Spectacularly, the level of serotonin in the feces of NAFLD rats was much higher than that in normal rats, while diosgenin weakened this alteration (Figure 4I). This phenomenon is worth exploring, and the detection of serum serotonin might be needed in future research.

Some biomarkers were likely to have much more biological significance than just being normal lipids or amino acid metabolites. The two most striking of them were dopamine and norepinephrine. Though these two neurotransmitters were very common in the pathophysiologic processes of diseases, their roles in NAFLD were rarely mentioned. This current study found the increases of both dopamine and norepinephrine in fecal samples of NAFLD rats. And diosgenin treatment down-regulated them remarkably (Figures 4A,G). Neurotransmitters routinely play roles on the central nervous system, especially in the brain. In consideration of the brain-gut axis, alterations of dopamine and norepinephrine were very likely to influence the brain. Additionally, modulation of gut microbiota on neurotransmitters has been recognized (Strandwitz, 2018). The correlation analysis of metabolites and gut microbiota also suggested their association. Two bacterial genera regulated by diosgenin showed significant correlations with dopamine and norepinephrine, all r values were greater than 0.7, and *p* values were less than 0.01.

In addition to lipid/amino acid metabolism related metabolites, this study also detected some other biomarkers. Though diosgenin might do not exhibit a satisfactory effect on these biomarkers. They deserved to be discussed for more roundly understanding the pathology or pathogenesis of NAFLD. Oral orotic acid is a common establishment method of NAFLD model, which proves its pivotal role in NAFLD occurrence (Wang et al., 2019; Jiang et al., 2021). Analogously, as a glucide, high sucrose could also promote the development of NAFLD (Lima et al., 2016; Gaballah et al., 2019). Orotic acid and sucrose were both detected to be elevated in NAFLD rats.

The metabolite changes detected in this study may also reflect the role of diosgenin on signaling pathways. According to literature reports, diosgenin could activate AMPK signaling and inhibit LXR signaling (Cheng et al., 2018). AMPK is a key enzyme in the regulation of biological energy metabolism, which activation could reprogram lipid metabolism in NAFLD rats (Garcia et al., 2019). AMPK activator was also reported to reduce BCAAs metabolic disorder in NAFLD mice (Binbin Zhang et al., 2021). Analogously, LXR had shown a strong correlation with lipid and amino acid metabolism (Ni et al., 2019). To sum up, the role of diosgenin on signaling pathways in the treatment of NAFLD is also worthy of attention.

## 5 Conclusion

**FIGURE 8:**
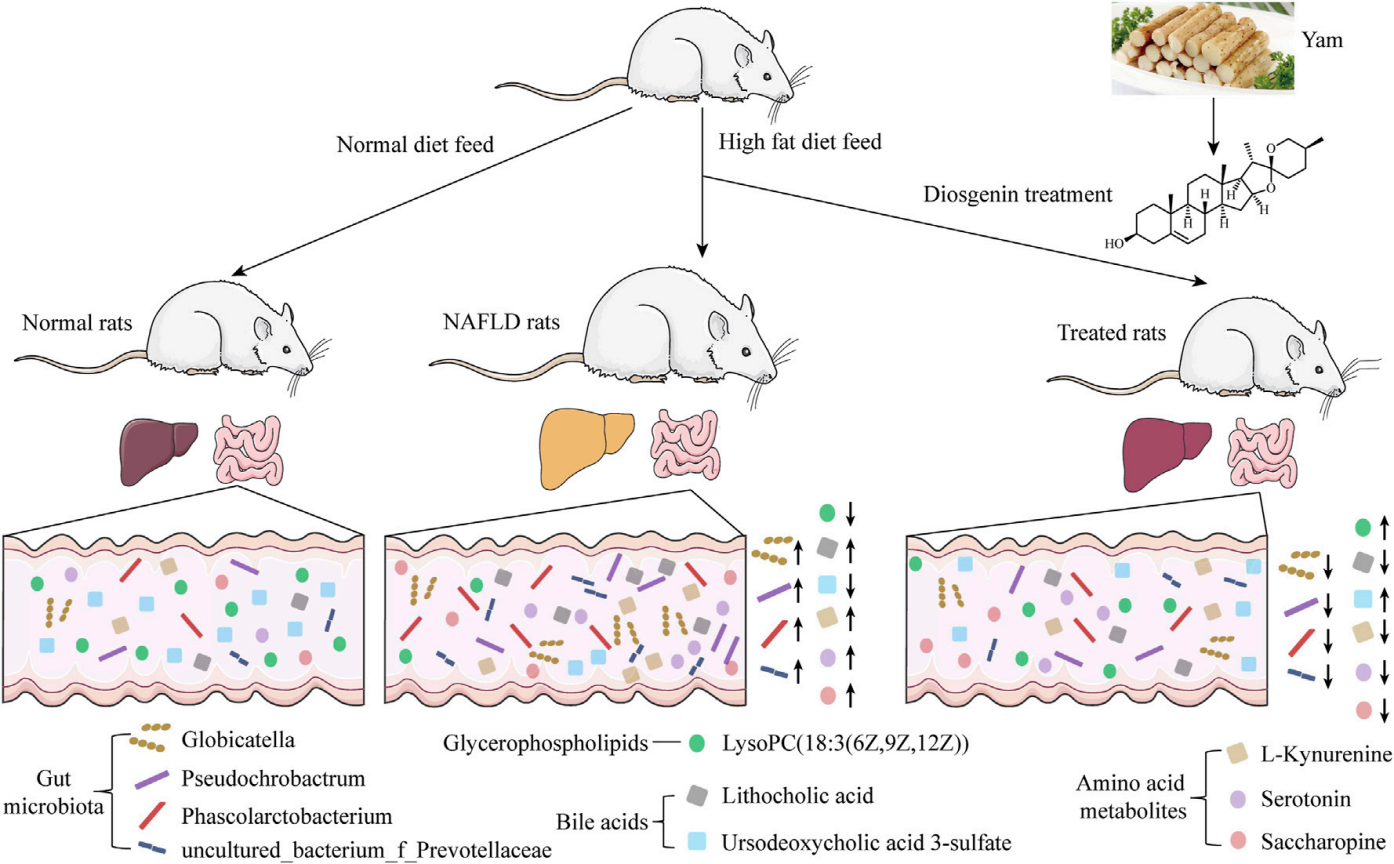
The function of diosgenin in high fat diet induced NAFLD rats.

Abnormal metabolism and gut microbiota disorder have been demonstrated to be involved in the occurrence and development of NAFLD. This study established a high-fat diet-induced NAFLD rat model and found diosgenin could reduce serum TC and TG levels, suppress excessive weight gain, and decrease fat accumulation in the liver of NAFLD rats. Fecal samples were selected to explore the alterations of metabolism and gut microbiota. As shown in Figure 8, diosgenin restored abnormal lipid and amino acid metabolism to a large extent. Down-regulation of lithocholic acid, up-regulation of ursodeoxycholic acid 3-sulfate, as well as effects on AAAs and lysine metabolism by diosgenin were impressive. Meanwhile, diosgenin improved the disturbance of gut microbiota, which also exhibited significant correlations with lipid and amino acid metabolism. Concretely, diosgenin decreased abnormally elevated *Globicatella, Phascolarctobacterium, Pseudochrobactrum, and uncultured_bacterium_f_*Prevotellaceae (Newgard et al., 2009; Xu et al., 2014; Cheng et al., 2015; Romero-Gomez et al., 2017; Jin Zhang et al., 2020; Asnicar et al., 2021; Mehmood et al., 2021).

## Data Availability

The original contributions presented in the study are publicly available. This data can be found here: https://www.ncbi.nlm.nih.gov/bioproject/PRJNA798160/.
