# Supplementary material for: Diosgenin Ameliorates Non-alcoholic Fatty Liver Disease by Modulating the Gut Microbiota and Related Lipid/Amino Acid Metabolism in High Fat Diet-Fed Rats
Source: Front Pharmacol. 2022 Apr 25;13:854790. doi: 10.3389/fphar.2022.854790 (PMC9081533; doi:10.3389/fphar.2022.854790)
Supplement: Supplementary file 1 [file DataSheet1.docx]

Supplementary Material

# Supplementary Figures


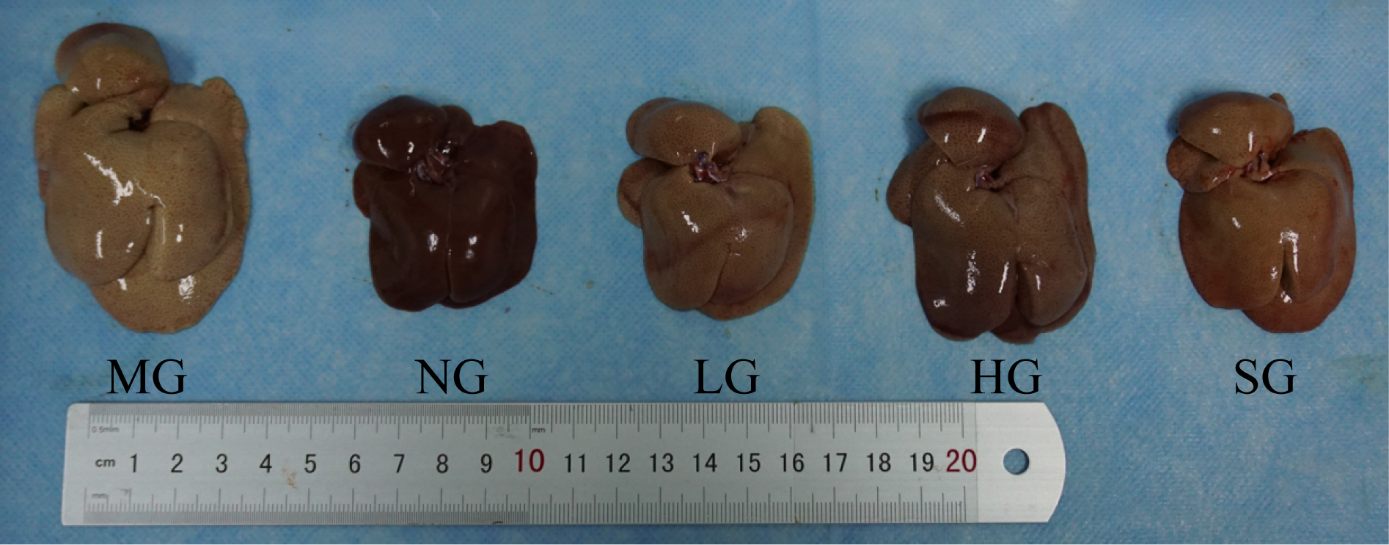


**Supplementary Figure 1.** Original macroscopic pictures of livers in MG, NG, LG, HG, and SG.

**
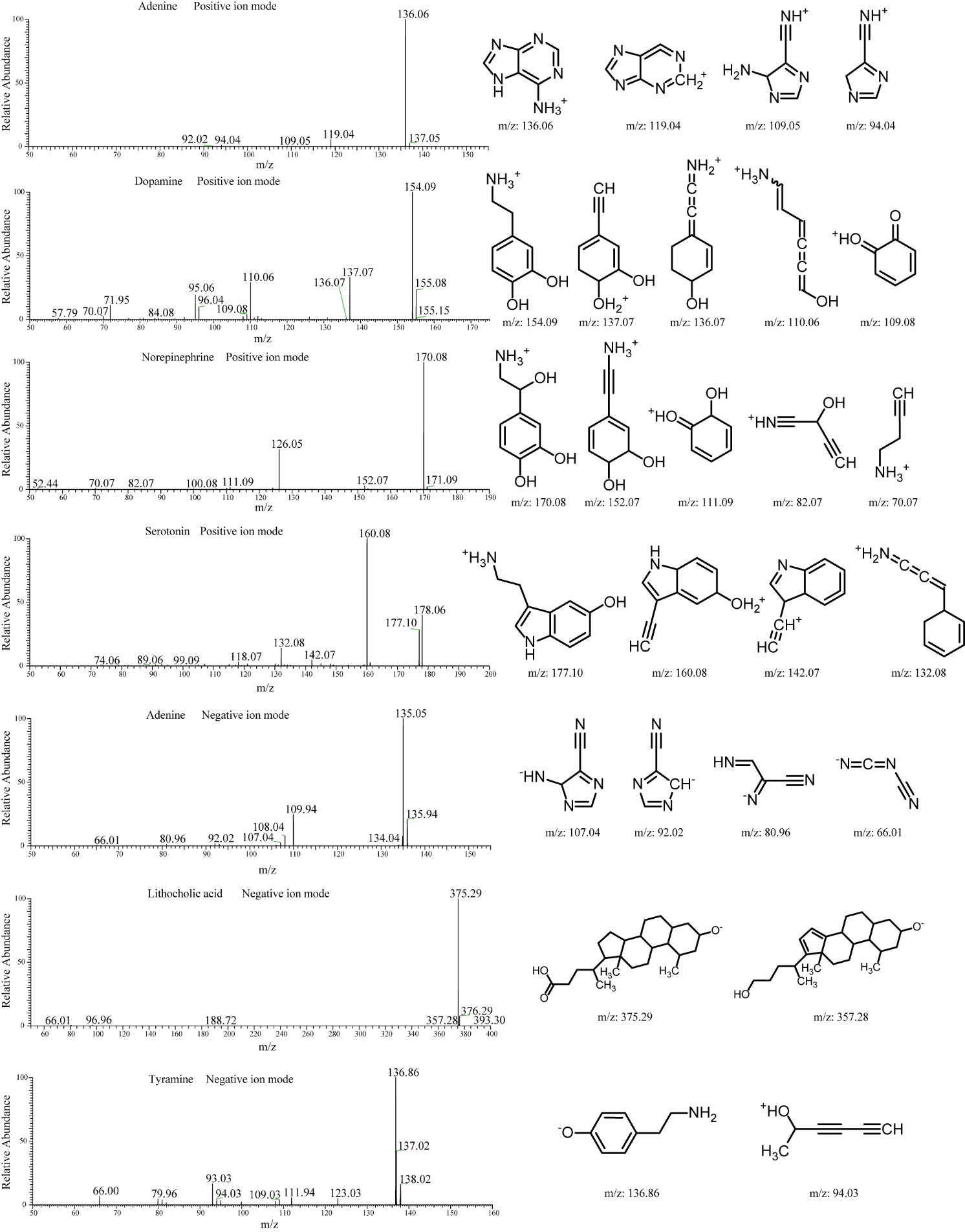
**

**Supplementary Figure 2.** MS/MS spectrums of metabolites and structural information of fragment ions.


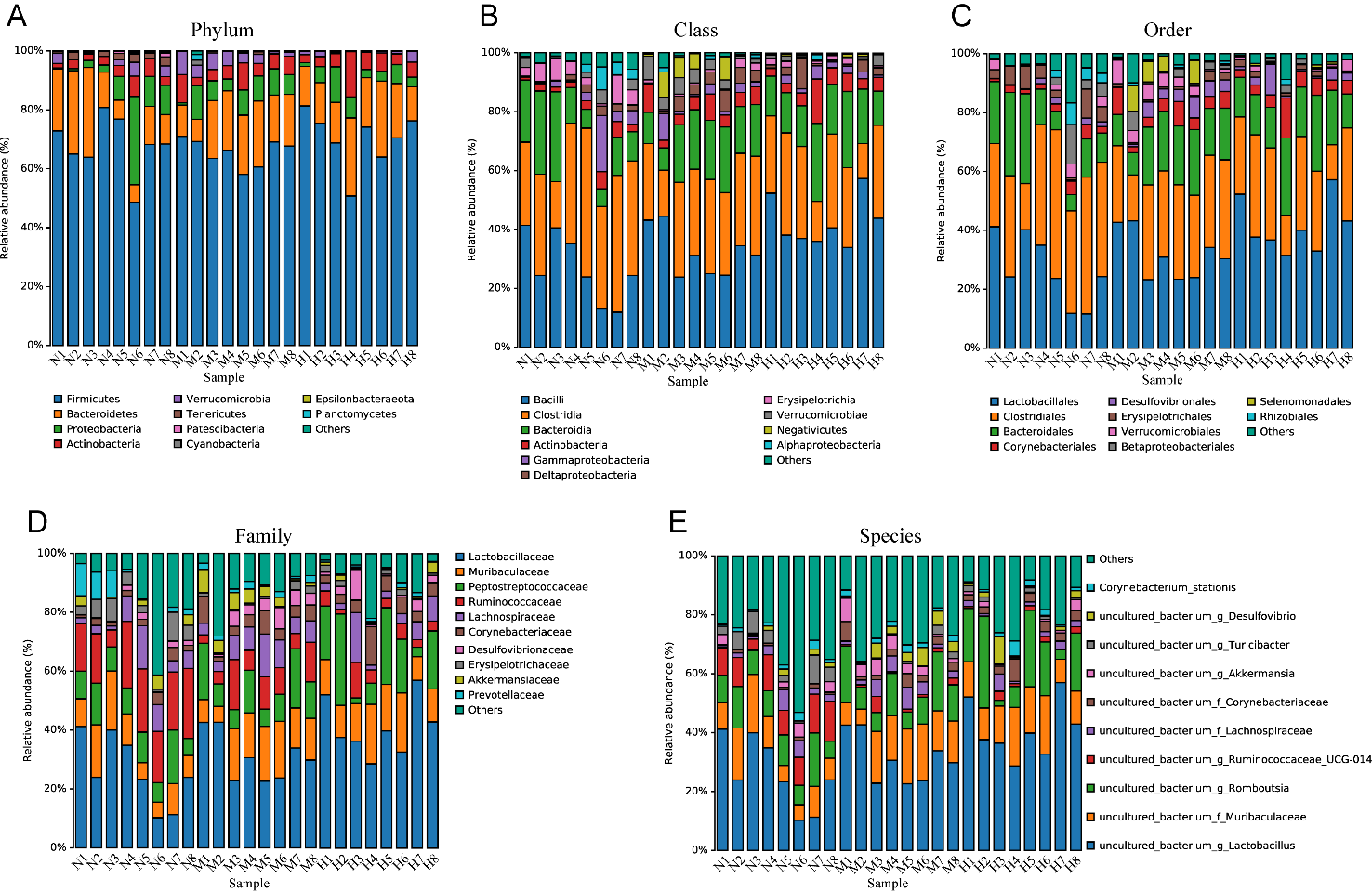


**Supplementary Figure 3.** Gut microbiota composition profile at levels of phylum **(A)**, class **(B)**, order **(C)**, family **(D)** and species **(E)**.
